# Plasma Levels of Preβ1-HDL Are Significantly Elevated in Non-Dialyzed Patients with Advanced Stages of Chronic Kidney Disease

**DOI:** 10.3390/ijms20051202

**Published:** 2019-03-09

**Authors:** Agnieszka Kuchta, Agnieszka Ćwiklińska, Monika Czaplińska, Ewa Wieczorek, Barbara Kortas-Stempak, Anna Gliwińska, Kamil Dąbkowski, Kornelia Sałaga-Zaleska, Agnieszka Mickiewicz, Alicja Dębska-Ślizień, Ewa Król, Maciej Jankowski

**Affiliations:** 1 Department of Clinical Chemistry, Medical University of Gdańsk, 80-210 Gdańsk, Poland; agnieszka.cwiklinska@gumed.edu.pl (A.Ć.); ewa.wieczorek@gumed.edu.pl (E.W.); barbara.kortas-stempak@gumed.edu.pl (B.K.-S.); anna.gliwinska@gumed.edu.pl (A.G.); kamil.dabkowski@gumed.edu.pl (K.D.); kornelia.salaga-zaleska@gumed.edu.pl (K.S.-Z.); maciej.jankowski@gumed.edu.pl (M.J.); 2 Clinic & Chair of Nephrology, Transplantology and Internal Diseases, Medical University of Gdańsk, 80-210 Gdańsk, Poland; monika.czaplinska@gumed.edu.pl (M.C.); alicja.debska-slizien@gumed.edu.pl (A.D.-Ś.); ewa.krol@gumed.edu.pl (E.K.); 3 1st Department of Cardiology, Medical University of Gdańsk, 80-210 Gdańsk, Poland; agnieszka.mickiewicz@gumed.edu.pl; 4 Laboratory of Molecular and Cellular Nephrology, Mossakowski Medical Research Centre Polish Academy of Sciences, 80-210 Gdańsk, Poland

**Keywords:** high-density lipoproteins, chronic kidney disease, atherosclerosis

## Abstract

In chronic kidney disease (CKD), the level of high-density lipoprotein (HDL) decreases markedly, but there is no strong inverse relationship between HDL-cholesterol (HDL-C) and cardiovascular diseases. This indicates that not only the HDL-C level, but also the other quantitative changes in the HDL particles can influence the protective functionality of these particles, and can play a key role in the increase of cardiovascular risk in CKD patients. The aim of the present study was the evaluation of the parameters that may give additional information about the HDL particles in the course of progressing CKD. For this purpose, we analyzed the concentrations of HDL containing apolipoprotein A-I without apolipoprotein A-II (LpA-I), preβ1-HDL, and myeloperoxidase (MPO), and the activity of paraoxonase-1 (PON-1) in 68 patients at various stages of CKD. The concentration of HDL cholesterol, MPO, PON-1, and lecithin-cholesterol acyltransferase (LCAT) activity were similar in all of the analyzed stages of CKD. We did not notice significant changes in the LpA-I concentrations in the following stages of CKD (3a CKD stage: 57 ± 19; 3b CKD stage: 54 ± 15; 4 CKD stage: 52 ± 14; *p* = 0.49). We found, however, that the preβ1-HDL concentration and preβ1-HDL/LpA-I ratio increased along with the progress of CKD, and were inversely correlated with the estimated glomerular filtration rate (eGFR), even after adjusting for age, gender, triacylglycerols (TAG), HDL cholesterol, and statin therapy (β = −0.41, *p* < 0.001; β = −0.33, *p* = 0.001, respectively). Our results support the earlier hypothesis that kidney disease leads to the modification of HDL particles, and show that the preβ1-HDL concentration is significantly elevated in non-dialyzed patients with advanced stages of CKD.

## 1. Introduction

A number of studies have shown that chronic kidney disease (CKD) is associated with high cardiovascular mortality as a result of accelerated atherosclerosis. An increased predisposition to atherosclerosis is largely driven by chronic inflammation, oxidative stress, and dyslipidaemia, which are usually associated with CKD [1,2,3]. The dyslipidemia of CKD patients is classically characterized by hypertriglyceridemia and a low concentration of high-density lipoprotein cholesterol (HDL-C) associated with normal or slightly reduced low-density lipoprotein cholesterol (LDL-C) levels [4,5]. Notably, although the serum HDL-C concentration exhibits a strong inverse association with cardiovascular disease in the general population, this association is attenuated and eventually abrogated as the kidney disease progresses [6]. This suggests that not only the cholesterol level of HDLs, but also the quality modification in HDL particles, may be crucial for the increase of cardiovascular risk in CKD patients. Several previous studies have shown that renal disorders may modify the concentrations of apolipoproteins, lipid transfer proteins, and enzymes connected with HDL particles, which may directly affect their biogenesis maturation, catabolism, and biological activity [7,8]. HDL represents a heterogeneous class of lipoproteins, whose particles may differ in composition and function. Because of the important role of apolipoproteins in maturation, metabolism, and functionality, the HDL particles are divided into those that do not contain apolipoprotein A-II (LpA-I) and those that contain the two main apolipoproteins, A-I and A-II (LpA-I/A-II).

Some clinical studies have suggested better athero-protective properties of LpA-I particles, although not all trials have supported these observations [9,10,11]. LpA-I, despite its apparent homogenous main apolipoprotein composition, is still a heterogeneous fraction, and its subfractions may differ both in size, density, and potentially in their antiatherogenic effects. One of the LpA-I subfractions—which, in recent years, has been of particular interest because of its important role in reverse cholesterol transport—is the preβ1-HDL fraction. Despite the fact that preβ1-HDL is known as an initial plasma acceptor of cell-derived cholesterol and plays an important role in the first step of reserve cholesterol transport, previous studies have shown the elevation of this HDL subfraction in patients with coronary artery disease, which may be connected with the delayed maturation of preβ1-HDL or its enhanced production [12,13]. A significant increase of the preβ1-HDL fraction was also observed in hemodialysis patients [14], but the data about non-dialysis patients are scarce.

Under normal circumstances, HDL particles protect against atherosclerosis through reverse cholesterol transport, but they also behave as an anti-oxidant, removing oxidant molecules from the arterial wall and limiting the oxidative modification of LDL [15]. CKD is a pathological state associated with the impaired antioxidative activity of HDL particles, possibly due to the reduced activity of HDL-associated enzymes, such as paraoxonase 1 (PON-1) and lecithin-cholesterol acyltransferase (LCAT). One of the potential pathways of losing the biological activity by HDL particles in CKD is an increase in myeloperosidase (MPO) activity in the course of the atherosclerotic processes in the wall of the human artery [16,17].

The aim of the present study was the evaluation of the parameters that may give additional information about HDL particles in the course of progressing CKD. For this purpose, we analysed the concentrations of LpA-I, preβ1-HDL, and MPO, and the activity of PON-1 and LCAT, in non-dialysis patients at various stages of CKD. In addition, we tested whether statin therapy has a significant impact on the qualitative and quantitative change of HDL subfractions.

## 2. Results

**Figure 1:**
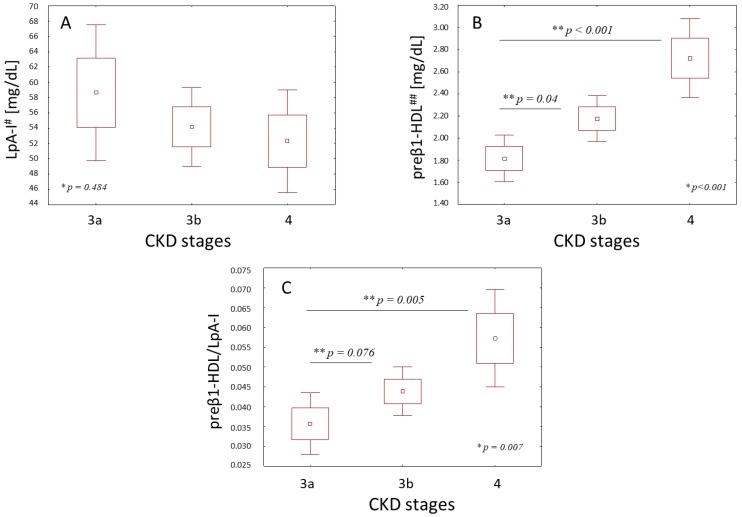
HDL containing apolipoprotein A-I without apolipoprotein A-II (LpA-I) (**A**), the preβ1-high-density lipoprotein (HDL) (**B**) concentrations, and the preβ1-HDL/LpA-I (**C**) values in the various stages of chronic kidney disease (CKD). Values are presented as mean ± standard error (SE) (2 SE) and were assessed using the analysis of variance (ANOVA) test * with the Tukey’s post-hoc test ** to determine where the differences existed; ^#^ ApoA-I content of LPAI; ^##^ ApoA-I content of preβ1-HDL.

The clinical characteristics of the patients at various stages of CKD are shown in Table 1. The analyzed groups did not differ in their average age, sex, and body mass index (BMI) index. The concentrations of HDL-C were similar in all of the analyzed stages of chronic kidney disease. The changes in the concentrations of triacylglycerols (TAG), total cholesterol (TC), and LDL-C were also insignificant between the study groups. The HDL particles from the patients at various stages of CKD did not differ in the concentrations of their main proteins—apolipoprotein A-I (ApoA-I) and apolipoprotein A-II (ApoA-II) (Table 1). The concentration of the HDL subpopulation containing apoA-I without apoA-II (LpA-I) decreased slightly in the following stages of CKD, but the changes were insignificant (Figure 1A). There was also no significant correlation between the LpA-I levels and the estimated glomerular filtration rate (eGFR) (Table 2). However, an analysis of the preβ1-HDL fraction showed its remarkable increase along with the progression of CKD (Figure 1B). The share of preβ1-HDL in the LpA-I fraction also increased significantly in the following stage of CKD (Figure 1C).

The univariate correlation analysis indicated that the preβ1-HDL concentration and preβ1-HDL/LpA-I ratio were inversely correlated with the severity of the CKD expressed by eGFR (Table 2), and remained an independent determinant of the CKD severity in the multiple linear regression after adjusting for age, gender, TAG, HDL-C, and statin therapy (Table 3).

The activity of LCAT was similar in all of the analyzed stages of chronic kidney disease (Table 1), and there were no significant correlations between LCAT activity and preβ1-HDL (R = −0.05; *p* = 0.968) or LpA-I concentration (R = 0.233; *p* = 0.062). No significant differences in the free cholesterol/total cholesterol ratio (FC/TC) in the course of the progression of CKD were also observed (Table 1).

As shown in Table 4, the authors did not observe a significant change in the PON-1 activity and MPO concentration in the following stages of CKD. The levels of both enzymes were also not correlated with each other (R = −0.57; *p* = 0.647).

**Figure 2:**
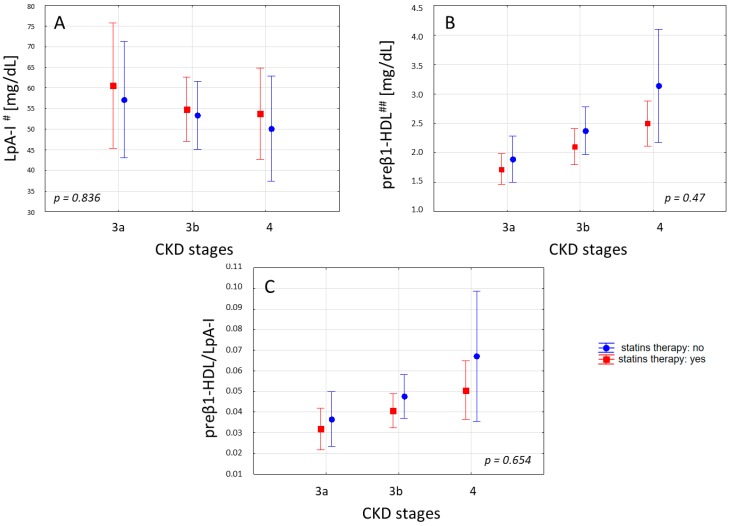
LpA-I (**A**), the preβ1-HDL (**B**) concentrations, and the preβ1-HDL/ LpA-I (**C**) values in the patients at various stages of CKD with and without statins therapy. Values are presented as mean ± 1.96 SE, and were assessed using the ANOVA test. ^#^ ApoA-I content of LPAI; ^##^ ApoA-I content of preβ1-HDL.

In the study groups, the statins were being taken by 41% (15% Atorvastatin (A.), 21% Simvastatin (S.), and 3% Rosuvastatin (R.)), 53% (35% A., 15% S., and 3% R.), and 64% (29% A. and 35% S.) of the patients in CKD stage 3a, 3b, and 4, respectively. We did not observe significant differences in the concentrations of HDL-C, apoA-I, apoA-II, and LCAT, and in the unesterified/total cholesterol ratio between the patients taking and not taking statins. Statins therapy has also no significant effect on the LpA-I, preβ1-HDL concentration, and preβ1-HDL/LpA-I ratio. However, we noted a tendency of a higher concentration of LpA-I, with a lower concentration of preβ1-HDL and preβ1-HDL/LpA-I ratio in the patients receiving statins in each of the analyzed groups of patients (Figure 2). Simultaneously, the significant differences in the preβ1-HDL concentration among the groups were maintained both in the patients receiving statins (*p* = 0.01) and those not receiving this drug (*p* = 0.005).

## 3. Discussion

The major finding of our study is the demonstration that the preβ1-HDL concentration increases in the 3a–4 stages of CKD. CKD is an independent cardiovascular risk factor, and the gradual decrease of eGFR in CKD patients translates into a linear increase of cardiovascular disease (CVD) mortality [18]. A number of clinical studies showed the increase of preβ1-HDL in patients with coronary artery disease, confirming the associations between increased levels of pre-preβ1-HDL and the risk of CAD, and proposed that high levels of preβ1-HDL remained a significant predictor of CVD even after adjustment for traditional risk factors [19,20,21]. Despite such an integrating role of the smallest HDL fraction in the atherosclerosis, the data about preβ1-HDL in non-dialysis patients with CKD are poor. Midda et al. [14] showed, however, a significant increase of preβ1-HDL in hemodialysis patients, and connected it with an impaired LCAT dependent conversion of preβ1-HDL to α-migrating HDL. Other clinical and experimental studies have also shown that CKD reduces the levels and activity of circulating LCAT, and down regulates the hepatic *LCAT* gene expression, especially in the advanced stages of the disease [22,23].

In a physiological condition, LCAT converts preβ1-HDL to α-migrating HDL, which transports the esterified cellular cholesterol to the liver for further processing. The impaired LCAT dependent catabolism of lipid poor HDL might be one of the reasons for the increased preβ1-HDL in patients with CKD. However, in our study, the change in LCAT activity along the progress of kidney disease was not significant. The FC/TC ratio, which confirms LCAT activity, was also invariable in the course of progressing CKD. We also did not observe any significant correlation between LCAT activity and preβ1-HDL concentration, which may suggest that the mechanism of the increase of preβ1-HDL levels in non-hemodialysis CKD patients might be complex and not only dependent on LCAT activity. The kidney is a major site of HDL homeostasis. Impaired renal catabolism of the immature small HDL particles may be another cause of preβ1-HDL elevation in patients with renal disorders. Additionally, as mentioned earlier, the elevation of the triglyceride-rich lipoprotein is typical for dyslipidemia in CKD. This induces the transfer of excess triglyceride into HDL particles and intensifies the conversion of α-migrating HDL to preβ1-HDL by cholesteryl ester transfer protein (CETP) activity. However, the multiple linear regression shows that the preβ1-HDL values remained an independent determinant of chronic kidney severity even after adjusting for TAG, age, gender, HDL cholesterol, and statin therapy (Table 3), which may confirm the hypothesis that the progressive disturbances in the HDL metabolism are one of the reasons for accelerated atherosclerosis in CKD patients. Some previous studies have suggested that the quantification of HDL particles containing ApoA-I without ApoA-II (LpA-I) could provide additional information in predicting coronary artery disease in the general population, although not all trials have supported this hypothesis [24]. To date, only a few studies have reported about the quantification of LpA-I in patients affected by renal disorders. Decreased levels of LpA-I were found in patients on continuous ambulatory peritoneal dialysis [25], however there are no data about LpA-I particles in non-dialysis patients. In our study, LpA-I decreased slightly in the following stages of CKD, but the change was insignificant (Figure 1A). There was also no significant correlation between LpA-I levels and the severity of kidney disease expressed by GFR (Table 2). When analyzing the share of preβ1-HDL in the LpA-I fraction, we show a clear increase of relative preβ1-HDL/LpA-I values as the GFR decreases (Figure 1C). These results suggest that renal disorders affect the distribution of HDL subfractions, and seem to be consistent with our earlier work in which we have shown that the distribution of LpA-I is different in patients with coronary artery disease [19]. However further research is required in order to test whether the observed quantitative changes in the HDL fractions reflect the dysfunctions of the functional proprieties of HDL particles in CKD patients.

Normal HDL particles possess potent antioxidant and anti-inflammatory properties that are as critical for protection against atherosclerosis as its role in reserve cholesterol transport. The antioxidant and anti-inflammatory properties of HDL are mediated, among others, by its constituent antioxidant enzyme—paraoxonase-1 (PON-1). PON-1 activity is an essential mechanism, by which HDL inhibits LDL oxidation. This enzyme hydrolyzes aromatic carboxylic acid esters, organophosphates, and oxidized phospholipids, simultaneously destroying biologically active lipids in mildly oxidized lipoproteins, thus protecting them against further oxidation [26]. CKD is a pathological state that alters the anti-oxidative and anti-inflammatory activity of HDL particles [27,28]. One of the potential pathways of converting an antiatherogenic HDL into its atherogenic forms in CKD involves a disturbance in the PON-1 activity caused by the pro-oxidant activity of myeloperoxidase (MPO) [28]. Growing evidence in the literature underscores the impact of the serum PON1 and MPO activity on the development of atherosclerotic changes in CKD patients [29,30].

In our study, we observed no significant change in the PON-1 and MPO activity along the progress of kidney disorders (Table 4). The activities of the enzymes were also not correlated with each other. However, an analysis of the earlier studies shows that significant changes in the activity of PON-1 and MPO appear most often in the end stages of chronic kidney disease [31,32], which may explain the lack of significance in our moderately advanced group of patients with CKD.

Of the patients, 51% had to undergo statin therapy in our study group. The importance of statin therapy in chronic kidney disease is not obvious. On the one hand, statins are generally considered to be effective in retarding CKD progression and reducing cardiovascular complication in patients with mild to moderate renal disease [5,33]. On the other hand, large clinical trials have failed to improve outcomes in patients with the end stage of the disease or maintenance dialysis [34,35,36].

The impact of statins on LpA-I subpopulations also remains in question. Some clinical studies have shown that statins decrease small preβ1-HDL and increase the concentration of the cholesterol-rich LpA-I fraction [25,37]. This effect is probably related to the inhibitory effect of statins on the activity of CETP, as well as the consequence of the lower number of lipoproteins available, sending triglyceride molecules in exchange for cholesterol from HDL. However not all clinical studies have shown that statins have a positive cardiovascular effect with more rapid maturation of small preβ1-HDL into cholesterol-rich α HDL [38,39]. In our study, we did not notice the significant impact of statin therapy on concentrations of LpA-I or preβ1-HDL. We observed only the tendency of a higher concentration of LpA-I and a lower concentration of preβ1-HDL in the patients receiving statins in the following stage of CKD, which maintained the significant differences in the preβ1-HDL concentration among the groups of the following CKD stages—both in the patients receiving statins and those not receiving this drug. However, considering the number of patients in our study groups, the influence of statins on the quantitative changes in the HDL factions requires a larger, and better matched, in terms of therapy, group of patients.

In conclusion, our results support an earlier hypothesis, that CKD leads to quantitative modification of HDL particles. The plasma levels of preβ1-HDL are significantly elevated in non-dialyzed patients with advanced stages of CKD. Further research is required in order to establish whether the preβ1-HDL concentration can serve as a biomarker of HDL quality, which can be used in the risk stratification and prognosis of cardiovascular complications in patients with CKD.

## 4. Materials and Methods

### 4.1. Subjects

Sixty-eight non-dialysis, adult patients from the Outpatient Clinic of the Nephrology Department of the Medical University of Gdańsk, in CKD stages 3a: GFR of 45–59; 3b: GFR of 30–44; and 4: GFR of 15–30 mL/min/1.73 m^2^, according to the four-parameter Modification of Diet in Renal Disease (MDRD 4-p) formula, were recruited into the study. The exclusion criteria were as follows: diabetes, proteinuruia in dipstick test and hypalbuminemia, liver diseases, malignancy, treatment with immunosuppressive agents, fibrates, or heparin preparations of acute diseases within three months before the study. The patients received the following statins: Atorvastatin (A.), Simvastatin (S.), and Rosuvastatin (R.), or they did not receive a lipid-lowering therapy. The study was performed in accordance with the ethical guidelines of the 1975 Declaration of Helsinki and was approved by the Medical Ethics Committee of the Medical University of Gdańsk (Project code: NKBBN/541/2014–2015; approved on 02 February2015). All of the participants provided written informed consent.

### 4.2. Laboratory Measurements

Blood samples were obtained between 07:00 and 08:00 following an overnight fast. The samples (serum or plasma) were separated after centrifugation at 1000× *g* for 15 min, and were stored at −80 °C pending analysis. For the preβ1-HDL test, we used plasma with edetic acid (EDTA). Immediately after sample collection, the blood was kept on ice and centrifuged within 15 min in ordr to separate the plasma, which was immediately diluted 21-fold with the stabilization buffer (50% sucrose and 0.05% NaN3). The stabilized sample was then stored at −80 °C. The total cholesterol (TC) and triacylglycerols (TAG) were measured using commercially available enzymatic kits obtained from Pointe Scientific (Warsaw, Poland). The free cholesterol (FC) was measured using an enzymatic kit obtained from Greiner Laboratories (Flacht, Germany).

The HDL was isolated by the precipitation of apolipoprotein B-containing lipoproteins with heparin and manganese chloride, and the HDL-cholesterol (HDL-C) was measured in supernatant using a kit obtained from Pointe Scientific. The LDL-cholesterol (LDL-C) level was calculated using the Friedewald formula.

The serum albumin was measured using the bromocresol green dye-binding method (Pointe Scientific). The ApoA-I and ApoA-II serum concentrations were determined using the nephelometric method with antibodies obtained from Siemens Healthcare Diagnostics (Escgborn, Germany) on a Behring laser nephelometer. The LPA-I concentration (ApoA-I content of LPA-I) was assayed using an electroimmunodiffusion technique (Hydragel LpAI, Sebia, Lisses, France).

The preβ1-HDL levels (ApoA-I content of preβ1-HDL) were measured according to a previously described method [40] by an enzyme immunoassay (preβ1-HDL ELISA; Sekisui Diagnostics, Lexington, MA, USA), which uses a mouse anti-human preβ1-HDL monoclonal antibody (MAb 55201) and a goat anti-human apoA-I polyclonal antibody. The LCAT activity was measured in the plasma using a commercial kit (MAK107-1KT, Sigma-Aldrich, Saint Luis, MO, USA), where the enzyme activity was evaluated as a ratio change of the fluorescence intensity of the substrate at 390 to 470 nm. The total paraoxonase-1 activity was measured in a serum with paraoxon ethyl as the substrate, according to the procedure described earlier [41]. Mieloperoxidase was analyzed in the plasma using an enzyme immunoassay kit (MPO ELISA; Immundiagnostik Bensheim, Gemany).

### 4.3. Statistics

The statistical analyses were performed using STATISTICA software, version 10 (StatSoft, Kraków, Poland). The Shapiro–Wilk test was used to test the determined normality of the distribution of variables. The continuous variables were expressed as mean ± SD (standard deviation), or as medians with 25th and 75th percentiles. The ANOVA or Kruskal–Wallis test were used to assess the differences between the groups with an appropriate post hoc test to determine exactly where the difference is significant. The Pearson’s chi-squared test was used to compare categorical variables. Univariate correlations were assessed using standardized Spearman coefficients. Multilinear regression was assessed using standardized β coefficients. P values below 0.05 were considered to be statistically significant.

## Figures and Tables

**Table 1:** Characteristics of patients at various stages of chronic kidney disease (CKD).

Parameter	Stages of CKD			
	3a	3b	4	*p*-Value
Gender (M/F)	12/5	19/15	12/5	0.531 **
Age (years)	69 ± 5	70 ± 9	63 ± 5	0.06 *
BMI (kg/m^2^)	28 ± 3	29 ± 5	26 ± 4	0.306 *
eGFR	50 ± 3	37 ± 4	22 ± 4	<0.001 *
Albumin (g/L)	43.6 ± 3.2	42.8 ± 2.9	42.9 ± 2.9	0.674 *
Statin therapy (%)	41	53	64	0.333 **
TAG (mg/dL)	102 ± 30	117 ± 45	135 ± 63	0.215 *
TC (mg/dL)	199 ± 42	200 ± 53	215 ± 36	0.345 *
HDL-C (mg/dL)	51 ± 11	50 ± 12	48 ± 11	0.761 *
LDL-C (mg/dL)	127 ± 39	127 ± 49	140 ± 33	0.335 *
ApoA-I (mg/dL)	172 ± 27	164 ± 27	160 ± 23	0.297 *
ApoA-II (mg/dL)	33 ± 7	31 ± 6	31 ± 5	0.499 *
LCAT (390/470 nm)	1.34 ± 0.03	1.32 ± 0.05	1.32 ± 0.04	0.458 *
FC/TC	0.283 ± 0.05	0.283 ± 0.03	0.290 ± 0.03	0.234 *

Continuous values are presented as means ± standard deviation (SD). Potential differences among the results were analysed using analysis of variance (ANOVA) * or the Pearson’s chi-squared test **. BMI—body mass index; eGFR—estimated glomerular filtration rate; TAG—triacylglycerols; TC—total cholesterol; HDL-C—high-density lipoprotein cholesterol; LDL-C—low-density lipoprotein cholesterol; ApoA-I—apolipoprotein A-I; ApoA-II—apolipoprotein A-II; LCAT—lecithin-cholesterol acyltransferase; FC/TC—free cholesterol/total cholesterol ratio.

**Table 2:** Univariate correlation between HDL-related parameters and eGFR.

Parameter	R	*p*
HDL-C (mg/dL)	0.104	0.382
ApoA-I (mg/dL)	0.153	0.202
ApoA-II (mg/dL)	0.109	0.358
LpA-I (mg/dL)	0.025	0.837
preβ1-HDL (mg/dL)	−0.456	<0.001
preβ1-HDL/LpA-I	−0.322	0.008
PON-1 (U/L)	0.068	0.566
MPO (ng/mL)	0.079	0.531
LCAT (390/470 nm)	0.080	0.484

R-Spearman’s correlation coefficient. PON-1—paraoxonase-1; MPO—myeloperoxidase.

**Table 3:** Multiple linear regression analysis for the eGFR value *.

Parameter	β	SE	*p*
preβ1-HDL	−0.41	0.105	<0.001
preβ1-HDL/LpA-I	−0.33	0.09	0.001

β standardized beta coefficients; SE—standard error; * adjusted for age, gender, statin therapy, HDL-C, and TAG concentration.

**Table 4:** Activity of paraoxonase-1 (PON-1) and myeloperoxidase (MPO) concentration.

	Stages of CKD			
	3a	3b	4	*p*-Value *
PON-1 (U/L)	102 (53–150)	83 (52–152)	113 (80–130)	0.890
MPO (ng/mL)	235 (136–392)	199 (139–347)	273 (160–327)	0.377

Values are presented as median (25th and 75th percentiles); * differences among the results were analyzed using the Kruskal–Wallis test.
